# Transparent reporting of recruitment and informed consent approaches in clinical trials recruiting children with minor parents in sub-Saharan Africa: a secondary analysis based on a systematic review

**DOI:** 10.1186/s12889-021-11079-y

**Published:** 2021-07-28

**Authors:** Angela De Pretto-Lazarova, Domnita Oana Brancati-Badarau, Christian Burri

**Affiliations:** 1 Swiss Tropical and Public Health Institute, Basel, Switzerland; 2 University of Basel, Basel, Switzerland; 3 Centre for Primary Health Care (uniham-bb), Liestal, Switzerland; 4 Aston University, Birmingham, UK

**Keywords:** Clinical trials, Informed consent, Children, Minor parents, Reporting, Sub-Saharan Africa

## Abstract

**Background:**

Standardised checklists of items to be addressed in clinical study protocols and publications are promoting transparency in research. However, particular specifications for exceptional cases, such as children with minor parents are missing. This study aimed to examine the level of transparency regarding recruitment and informed consent approaches in publications of clinical trials recruiting children with minor parents in sub-Saharan Africa. We thereby focused particularly on the transparency about consenting persons (i.e. proxy decision-makers) and assessed the need to expand reporting guidelines for such exceptional cases.

**Methods:**

We conducted a secondary analysis of clinical trial publications previously identified through a systematic review. Multiple scientific databases were searched up to March 2019. Clinical trial publications addressing consent and potentially recruiting children with minor parents in sub-Saharan Africa were included. 44 of the in total 4382 screened articles met our inclusion criteria. A descriptive analysis was performed.

**Results:**

None of the included articles provided full evidence on whether any recruited children had minor parents and how consent was obtained for them. Four proxy decision-maker types were identified (parents; parents or guardians; guardians; or caregivers), with further descriptions provided rarely and mostly in referenced clinical trial registrations or protocols. Also, terminology describing proxy decision-makers was often used inconsistently.

**Conclusions:**

Reporting the minimum maternal age alongside maternal data provided in baseline demographics can increase transparency on the recruitment of children with minor mothers. The CONSORT checklist should require clinical trial publications to state or reference exceptional informed consent procedures applied for special population groups. A standardized definition of proxy decision-maker types in international clinical trial guidelines would facilitate correct and transparent informed consent for children and children with minor parents.

**Study registration:**

CRD42018074220.

**Supplementary Information:**

The online version contains supplementary material available at 10.1186/s12889-021-11079-y.

## Background

Children under 5 years in sub-Saharan Africa (SSA) are disproportionately affected by malnutrition and infectious diseases [1, 2], which contributes to a higher percentage of paediatric clinical trials (CTs) performed in SSA compared to Europe or the US [3, 4]. As teenage pregnancy rates in SSA are among the highest worldwide [5], researchers will likely encounter children with minor parents when conducting CTs in this region [6–8].

Previous evidence from CTs in low- and middle-income countries (LMICs) indicates that researchers face particular challenges when implementing informed consent (IC) for children with minor parents. The legal status of minor parents and their ability to consent for their children is often unclear [9]. The appropriate consenting person (i.e. proxy decision-maker^1^) in this case may vary depending on local legal and cultural conditions. Sometimes also ad hoc solutions tailored to local customs might be implemented [7, 10]. However, despite the need for careful ethical considerations for this research group [11], evidence on practices is scarce and described typically in secondary studies, if at all [9].

Ethical guidance in research requires not only that consent is provided, but that it is documented and reported transparently [12], which promotes public confidence in research [13]. As insufficient transparency can contribute to biased and incomparable research publications, it was found to be a significant source of “waste” in the conduct of research [14]. Hence, CT registration and publication of CT protocols, results, and participant-level datasets have been widely promoted to increase the usefulness and value of CT documentation [15]. For a more consistent and complete availability of CT information, standardised checklists of critical items to be addressed in CT protocols and publications were implemented [16, 17]. The Consolidated Standards of Reporting Trials (CONSORT) statement guides the CT publications’ content. It also includes requirements for details on CT registration and access to study protocols. The Standard Protocol Items: Recommendations for Interventional Trials (SPIRIT) statement guides protocol contents. The endorsement of reporting guidelines contributed to an improved reporting quality of CTs over time [18].

Paediatric CTs require special considerations and researchers have argued for a specific checklist with additional reporting items for children. A recommended CONSORT adaptation for children was published in 2010 [19]. A different, evidence-based extension of the CONSORT checklist for children and a first extension of the SPIRIT checklist for children are currently being developed [20]. However, in the published development steps, neither of these checklists addresses unique circumstances encountered more frequently in LMICs, such as children with minor parents.

We conducted a systematic literature review including any type of literature to gain evidence on how IC is provided for children with minor parents in SSA. In a primary analysis published elsewhere [9], we focused on publications providing evidence about children recruited in CTs having minor parents and/or the respective consent process. While our search also retrieved CT publications, none were eligible for the primary analysis since they all lacked such evidence. Nevertheless, the identified CT publications represented a sample of studies potentially involving children with minor parents and provided the opportunity to investigate specific gaps and transparency in reporting details on the recruitment and consent processes implemented for such children. Therefore, we conducted a secondary analysis of this sample of CT publications, aiming to determine the level of transparency relating to CT participation of children with minor parents and the types of proxy decision-makers providing consent.

Childhood diseases lacking specific paediatric therapies are often treated with medicines used outside their labelling indications. Therefore, CTs collecting data on the safety and efficacy of medicines in children, particularly on off-label use, are essential to prevent children from receiving ineffective therapies with unknown and likely harmful adverse drug reactions [21]. However, IC in paediatric research, and particularly in children with minor parents, is complex. The way it is reported deserves specific attention to avoid inconsistent, unethical, and improper approaches that could be used in CTs. The complexity of obtaining consent for such infants may unintentionally lead to their exclusion from research resulting in a lack of data for this highly vulnerable group, which will have long-term implications for their health.

## Methods

We carried out a systematic literature review registered in the PROSPERO database (CRD42018074220) [22]. All the criteria and terms guiding this review were pre-defined, but no protocol was published.

This secondary analysis was based on the same search strategy and screening steps as the primary analysis [9], but we used different eligibility criteria (see Eligibility criteria) and performed separate full-text assessments.

### Search strategy

We searched PubMed/MEDLINE, Embase, CINAHL, and Google Scholar without any time limitation. The search strategy included the elements of IC, decision-making, CTs, minors, and SSA. We performed the first search in July 2017 and updated it in March 2019 based on a revised and improved search strategy [9]. The references of included articles were not systematically searched. However, for publications that explicitly stated that some methodology details were published in other articles, we considered secondary sources for the analysis. We did not list the secondary articles separately in the results, but included them as supplementary data accompanying primary CT publications.

### Eligibility criteria

Articles were included if they were publications of CTs involving children in SSA whose parents were potentially minors. Minor parents were broadly defined as adolescents between the age of 12 and the respective age of majority in each country, who are the parents of a CT child participant. If information on the parental age was not given, we considered studies in which child participants were < 5 years, because for this age group the probability of minor parents is higher [23]. We referred to CTs as prospective health-related interventions in persons [24]. Health-related interventions included drug, vaccine, diagnostic, medical device, surgical, emergency research, and dietary supplements trials. CTs had to have taken place in at least one SSA country. We only included publications in English or French.

### Data extraction and analysis

The search results were imported into the reference management software Endnote X7. After removing duplicates, we extracted information (Author, Year, Journal/Publisher, Title, Abstract, Keywords, ISBN/ISSN, DOI, and URL) into an MS Excel table for screening. The identified articles were screened in two steps: First, two independent reviewers (ADP and DOB) screened titles and abstracts for potentially eligible articles. Second, if information about an inclusion criterion was missing, one reviewer (ADP) screened additionally full-texts. The reviewers reached a moderate (Cohen’s Kappa *κ* = 0.47) and substantial (Cohen’s Kappa *κ* = 0.71) agreement for the title and abstract screening of the initial and updated search, respectively [25]. Disagreements were mostly systematic, mainly concerning the distinction of study types, and were all resolved through discussion. One researcher (ADP) then assessed the full-texts of potentially included articles for final eligibility and extracted data. The second reviewer (DOB) crosschecked 10% of the full-text articles for eligibility and the extracted information.

Extracted data of resulting full-text articles included CT characteristics and the following elements for analysis: Study location, health condition, medical intervention, population size and type, CT design, ethics committee (EC)/institutional review board (IRB) approval, information sections addressing eligibility criteria, IC approach, and proxy decision-makers. Whenever applicable, we accessed the referenced regulatory and ethical guidance, CT registration details, supplementary files, and referenced protocols. We considered specific sections of the CONSORT statement [17], and its adaptation for children [19] to identify information concerning the recruitment and IC approach for children with minor parents. The considered CONSORT sections included the eligibility criteria, participant flow diagram (exclusions), baseline data, ethical considerations, and access to protocol and registry information. We performed a descriptive analysis using MAXQDA (VERBI GmbH) and MS Excel.

In order to assess transparency on reported proxy decision-makers, we defined three levels of transparency according to the level of detail of their description: A basic level of transparency was assigned when neither the type nor the number of proxy decision-makers was specified. A first level of extended transparency was defined when the type or number of proxy decision-makers was specified. A second level of extended transparency was attributed when also the proxy decision-makers’ age or competence was taken into account.

## Results

**Fig. 1:**
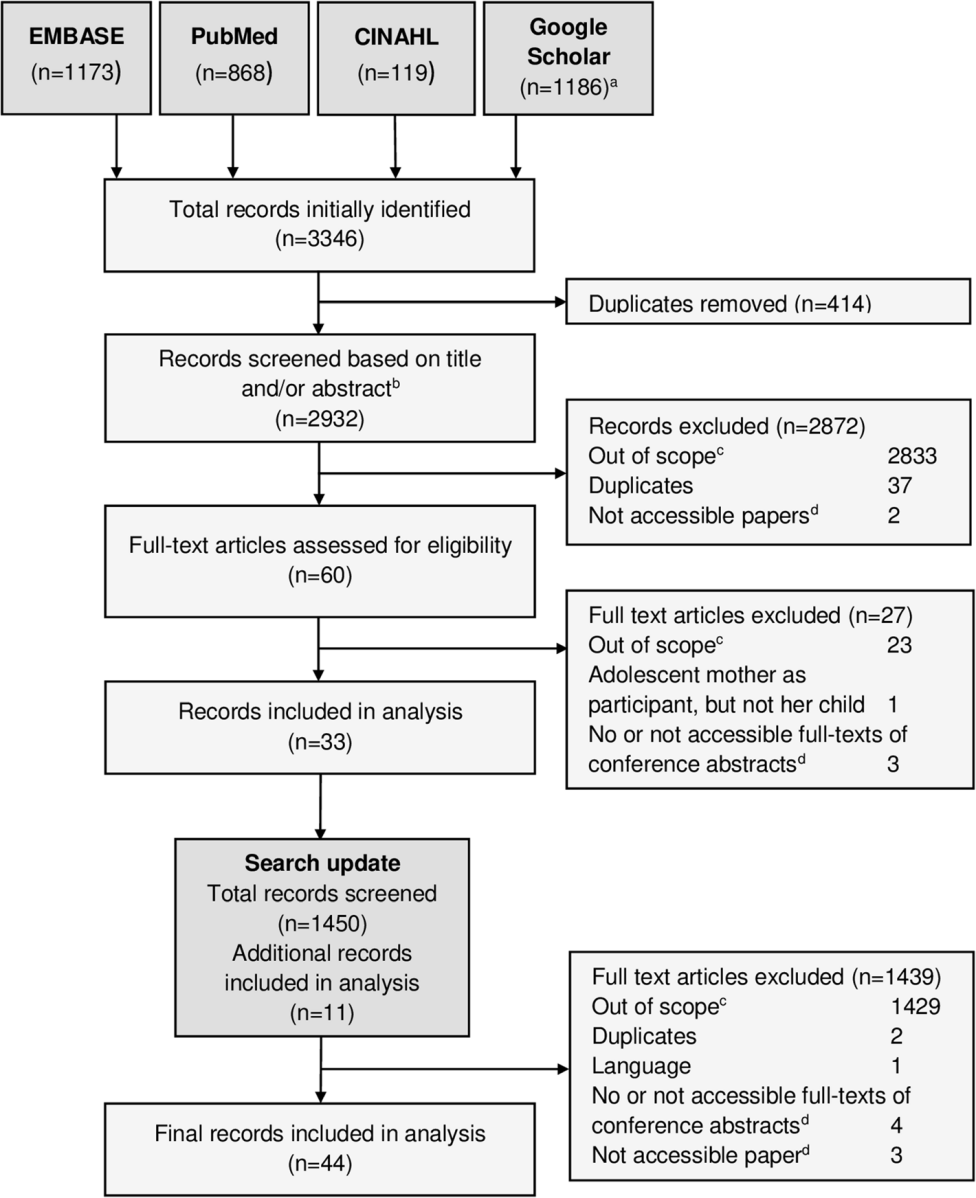
Study-selection flow diagram. ^a^ Total number results from three combined Google Scholar searches. ^b^ If the title or abstract lacked information on key elements of the search, the full text of the articles was also screened. ^c^ Out of scope: not addressing SSA, not addressing children < 5, clearly addressing adult parents, and not being a clinical trial publication. ^d^ List of full-texts that could not be accessed or found is provided in the additional material (see Additional file 1, Table S1)

A total of 4382 screened articles met our inclusion criteria. In an initial search, we identified 3346 articles (Fig. 1). After removing duplicates (*n* = 414), 2932 articles were screened, of which 2872 articles were excluded. The full-text was assessed for 60 articles, resulting in 33 included publications. A search update identified 1450 additional articles, from which 11 were eligible. In total, 44 articles were included in the analysis.

The included articles’ publication dates ranged from 1990 to 2017. More than half of them (*n* = 25/44, 56.8%) were published from 2011 onward (Table 1). Two of the included articles were conference abstracts [26, 51]. The CTs were conducted in 17 different countries in SSA, with Malawi mentioned most frequently (*n* = 8), followed by Ghana (*n* = 6) and South Africa (n = 6). Malaria (*n* = 15) followed by undernutrition (*n* = 7), and rotavirus gastroenteritis (*n* = 5) were the most frequently addressed health conditions, while antimalarials (*n* = 14), followed by dietary supplements (*n* = 10) and vaccines (n = 10) were the most reported interventions. Included study participants were mostly infants only (*n* = 17/44, birth to < 2 years), or infants and children combined (*n* = 17/44, birth to < 12 years). Five studies also enrolled adolescents (12 to < 18 years), four also included adults (18 years and over), and one addressed only children (*n* = 1/44, 2 to < 12 years). 31 of the 44 publications provided the possibility of stratification according to the participants’ age and included a total of 75,063 children under 5 years.

**Table 1:** Study Characteristics

#	Author	Country	Health condition	Intervention	Study Population size	Study population age range
1	Achonduh et al. (2012) [26]	Cameroon	Malaria	Dietary supplements (vitamin A, zinc) and antimalarials (artesunate-amodiaquine)	100	6–24 months
2	Adegbehingbe et al. (2010) [27]	Nigeria	Clubfoot	Surgical methods (Ponseti method and extensive soft tissue surgery)	105	0–adult
3	Afolabi et al. (2013) [28]	The Gambia	HIV	Vaccine	48	0–9 months
4	Aluka et al. (2013) [29]	Nigeria	Fever	Coldwater sponging, oral paracetamol	88	12–120 months
5	Amadi et al. (2002) [30]	Zambia	Diarrhoea and malnutrition (cryptosporidiosis)	Antiparasitic (nitazoxanide)	96	1–7 years
6	Arimond et al. (2017) [31]	Ghana, Malawi, Burkina Faso	Undernutrition	Dietary supplements (lipid-based)	2622, 1318, 1093, 625	0–18 months
7	Armah et al. (2010) [32]	Ghana, Kenya, Mali	Gastroenteritis (Rotavirus)	Vaccine	5468	4–12 weeks
8	Armah et al. (2013) [33]	Ghana	Gastroenteritis (Rotavirus)	Vaccine	998	0–29 days
9	Christofides et al. (2006) [34]	Ghana	Anaemia (Iron deficiency)	Dietary supplement (iron)	133	6–18 months
10	Corbett et al. (2010) [35]	Malawi	HIV	Antiretrovirals (lamivudine, stavudine, nevirapine)	18	1–13 years
11	Egere et al. (2012) [36]	The Gambia	Pneumonia (*Streptococcus pneumoniae*)	Vaccine	328	2–30 months
12	Gilliams et al. (2014) [37]	Malawi	Malaria	Antimalarials (chloroquine-azithromycin)	320	20–46 months
13	Goodhew et al. (2014) [38]	Tanzania	Trachoma	Mass drug administration (azithromycin)	264	1–6 years
14	Hassall et al. (2015) [39]	Kenya	Malaria	Umbilical cord red blood cell transfusion	55	0–6 years
15	Hess et al. (2015) [40]	Burkina Faso	Undernutrition (Growth stunting)	Dietary supplements (lipid-based)	3220	9 months
16	Hesseling et al. (2005) [41]	Malawi	Burkitt Lymphoma	Chemotherapy (vincristine, methotrexate, leucovorin, cyclophosphamide, prednisone)	60	3–16 years
17	Hussey et al. (1990) [42]	South Africa	Measles	Dietary supplement (vitamin A)	189	0–13 years
18	Isanaka (2017) [43]	Niger	Gastroenteritis (Rotavirus)	Vaccine	3508	6–14 weeks
19	Kone et al. (2010) [44]	Mali	Malaria (Glucose-6-phosphate dehydrogenase deficiency)	Antimalarials (artemether-lumefantrine, artesunate-mefloquine)	315	> 1 year
20	Koram et al. (2005) [45]	Ghana	Malaria	Antimalarials (amodiaquine-artesunate, artemether-lumefantrine, sulfadoxine-pyrimethamine, chloroquine)	168	6–59 months
21	Madhi et al. (2011) [46]	South Africa	Childhood diseases (Hepatitis B, diphtheria, tetanus, pertussis, polio, Haemophilus influaenzae)	Vaccines	715	0–3 days
22	Madhi et al. (2012) [47]	South Africa, Malawi	Gastroenteritis (Rotavirus)	Vaccine	3168	6–16 weeks
23	Maka et al. (2015) [48]	Cameroon	Malaria	Antimalarials (artesunate, quinine)	238	3 months–15 years
24	Mangani et al. (2015) [49]	Malawi	Undernutrition (Growth stunting)	Dietary supplements (lipid-based, corn-soy blend)	840	5.5–6.5 months
25	Meremikwu et al. (2006) [50]	Nigeria	Malaria	Antimalarials (artemether-lumefantrine, artesunate-amodiaquine)	119	6–59 months
26	Meremikwu et al. (2016) [51]	Nigeria	Malaria	Antimalarials (artesunate-amodiaquine, dihydroartemisinin-piperaquine, artemether-lumefantrine)	493	6–59 months
27	Michael et al. (2010) [52]	Nigeria	Malaria	Antimalarials (artemether-lumefantrine, artesunate-amodiaquine)	193	12–132 months
28	Ngasala et al. (2011) [53]	Tanzania	Malaria	Antimalarials (artemether-lumefantrine)	300	3–59 months
29	Nji et al. (2015) [54]	Cameroon	Malaria	Antimalarials (dihydroartemisin-piperaquine, artesunate-amodiaquine vs artemether-lumefantrine)	720	6 months–10 years
30	Nwanyanwu et al. (1996) [55]	Malawi	Malaria	Antimalarials (sulphadoxine-pyrimethamine)	159	0–5 years
31	Phuka et al. (2008) [56]	Malawi	Undernutrition (Growth stunting)	Dietary supplements (fortified spread, micronutrient-fortified maize-soy flour)	182	6–18 months
32	Rahimy et al. (1999) [57]	Benin	Fever (in Sickle Cell Disease)	Antibiotics (outpatient management)	61	0–12 years
33	Robertson et al. (2011) [58]	Uganda	Perinatal asphyxial encephalopathy	Therapeutic hypothermia	36	3 h
34	Roca et al. (2011) [59]	The Gambia	Pneumococcal disease	Vaccine	5441	0–adult
35	Sazawal et al. (2007) [60]	Zanzibar	Undernutrition (Mortality)	Dietary supplement (zinc)	42,546	1–36 months
36	Schellenberg et al. (2001) [61]	Tanzania	Malaria and anaemia	Antimalarials (sulphadoxine-pyrimethamine) alongside routine vaccinations	701	0–1 year
37	Singana et al. (2016) [62]	Republic of Congo	Malaria	Antimalarials (artesunate-amodiaquine, artemether-lumefantrine)	198	< 12 years
38	Sissoko et al. (2016) [63]	Guinea	Ebola	Antiviral (favipiravir)	111	> 1 year
39	Sow et al. (2012) [64]	Mali	Gastroenteritis (Rotavirus)	Vaccine	1960	48 days (median age)
40	Te Water Naude et al. (2000) [65]	South Africa	Tuberculosis	Chemotherapy (isoniazid, rifampin, pyrazinamide)	206	0–14 years
41	The Zinc Against Plasmodium Study Group (2002) [66]	Ecuador, Ghana, Tanzania, Uganda, Zambia	Malaria	Antimalarial and dietary supplement (chloroquine and zinc)	1087	6–60 months
42	Urban et al. (2008) [67]	South Africa	Nutrition (Infant growth)	Dietary supplements (biologically acidified milk, probiotics)	85	0–1 week
43	Waggie et al. (2011) [68]	South Africa	Polio	Vaccine	800	0–30 days
44	Yohannan et al. (2013) [69]	Tanzania	Trachoma	Mass drug administration (azithromycin, tetracycline)	2261	0–5 years

### General reporting characteristics

Table 2 summarises general reporting characteristics relating to the research design, EC/IRB approval, implementation of regulatory or ethical guidance, supplementary material (i.e. CT registrations, protocols and supplementary files), and the section addressing informed consent (IC).

**Table 2:** General Reporting Characteristics

Characteristics	n (Total ***n*** = 44)	(%)
**CT design**		
Randomised, controlled (including cluster- and community-randomised)	37	(84.1)
* Control*		
Placebo-controlled	12	(32.4)
Treatment-controlled	24	(64.9)
No treatment-controlled	1	(2.7)
* Blinding*		
Blinded (including double-, single-, and partially-blinded)	21	(56.8)
Open-label	10	(27.0)
NDA	6	(16.2)
* Phase*		
Phase I	1	(2.7)
Phase III	3	(8.1)
NDA	33	(89.2)
Non-randomised, single-arm	6	(13.6)
NDA	1	(2.3)
**EC/ IRB approval**		
By multiple national and external ECs/IRBs (local regulatory authorities, local IRBs, national ECs, international ECs, external national ECs, and external IRBs)	24	(54.5)
By multiple local (local regulatory authorities, local IRBs, national ECs)	1	(2.3)
Only by local EC/IRB	13	(29.5)
Only by national EC	1	(2.3)
NDA	5	(11.4)
**Regulatory/ethical guidance**^**a**^		
Good Clinical Practice	13	(29.5)
Declaration of Helsinki	9	(20.5)
National/local regulatory requirements	7	(15.9)
Good Laboratory Practice	1	(2.3)
n/a (Conference abstract)	2	(4.5)
NDA	27	(61.4)
**Supplementary material**^**a**^		
CT registration^b^	21	(47.7)
CT publications including supplementary files	9	(20.5)
Protocol as supplementary file^c^	6	(13.6)
**CT publication sections and files addressing IC**^**a**^		
Abstract	18	(40.9)
Methods	41	(93.2)
Eligibility	27	(65.9)
Ethics	34	(82.9)
Results	15	(34.1)
Discussion	1	(2.3)

*CT* Clinical trial, *EC* Ethics committee, *IRB* Institutional review board, *NDA* No data available, *IC* Informed consent

^a^ Numbers do not add up, since several features may apply and some publications were inconclusive in the description

^b^ 60% of publications since CT registration became a requirement by the ICMJE in 2005 [70]

^c^ 24% of publications since protocol publication became a requirement by the CONSORT statement in 2010 [17]

### Transparency of CT recruitment of children with minor parents

Information on maternal age could potentially be found in publications among the description of the eligibility criteria, the effective exclusion reasons in study flow diagrams, and the baseline data about study participants. While in most CTs, children were directly recruited, in four CTs, recruitment was first based on the eligibility of pregnant women [31, 33, 67, 68]. One of these four publications [31] referred to a CT conducted in Malawi recruiting mothers and children explicitly as a dyad [71]. It was the only CT publication addressing minor mothers in the eligibility criteria. Mothers from 15 years of age were considered eligible, while the legal age of majority in Malawi was 18 at the time [72]. However, there is no evidence in any section of the CT publications that any mother meeting this criterion actually was recruited and participated in the CT: Neither the exclusion reason in the study flow diagram, which stated “underage”, nor the way the maternal age was described in the baseline data (i.e. only mentioning the mean age, including standard deviation without explicitly stating the minimal age of included mothers) were sufficient to allow such a confirmation. Two more CT publications described maternal age in the same way in the baseline data [40, 67], and no other CT publication provided details about parental age in the main text.

### Transparency on proxy decision-makers for children’s CT participation

Proxy decision-makers providing IC for children’s CT participation were mentioned 77 times across the CT publications or supplementary materials (i.e., CT registration, protocol, or referenced articles further detailing the methodology) (Table 3). We found the terminology used to describe proxy decision-makers to be variable and identified four main types: “parents” (39.0%); “parents or guardians” (36.4%); “caregivers” (13.0%); and “guardians” (11.7%). Further details were provided in some CT publications or supplementary material specifying a subtype and number of proxy decision-makers. Subtypes were specified in a third (32.5%), and the number was specified in about half (50.6%) of all cases. In 40.3% of all cases, neither the subtype nor the number was specified.

**Table 3:** Proxy decision-maker types, subtypes, and numbers mentioned across CT publications and supplementary material

Proxy decision-maker description	n	(%)
Total	77	(100.0)
**Parents**	**30**	**(39.0)**
Unspecified parents	23	(29.9)
- Unspecified number	15	(19.5)
- One^a^	8	(10.3)
Mother	4	(5.2)
Mother involving fathers/partners/husbands involved in the decision-making	1	(1.3)
Mother able to understand study procedures and give consent/of a specific age	2	(2.6)
**Parents or guardians**	**28**	**(36.4)**
Unspecified parents or guardians	21	(27.3)
- Unspecified number	13	(16.9)
- One^a^	6	(7.8)
- At least one	1	(1.3)
- Each	1	(1.3)
Parents or legal guardians/legally acceptable representatives	4	(5.2)
- Unspecified number	2	(2.6)
- One^a^	1	(1.3)
- Each	1	(1.3)
Parents or guardians of legal age/adult/with the ability to give informed consent	3	(3.9)
- Unspecified number	2	(2.6)
- One^a^	1	(1.3)
**Caregivers (incl. Caretaker, carer)**	**10**	**(13.0)**
Unspecified caregivers	5	(6.5)
- Unspecified number	3	(3.9)
- One^a^	1	(1.3)
- At least one	1	(1.3)
Family	3	(3.9)
- Unspecified number	3	(3.9)
Primary caregiver (Mother/Father/Legal guardian)	2	(2.6)
- One^a^	2	(2.6)
**Guardians**	**9**	**(11.7)**
Unspecified guardians	3	(3.9)
- One^a^	2	(2.6)
- At least one	1	(1.3)
Authorised/identifiable/legal guardian	5	(6.5)
- One^a^	3	(3.9)
- At least one	2	(2.6)
Guardian capable of providing consent	1	(1.3)
- One^a^	1	(1.3)

^a^ Single proxy decision-makers were counted whenever the singular was employed to refer to consent by a parent/guardian. When consent was provided by mothers with no other specification, only one decision-maker was counted. Otherwise, terms in their plural form, as well as the designation of “parental” were recorded as “unspecified number”, unless other indications regarding multiple consenters were provided

The terms used to describe proxy decision-makers were sometimes inconsistent within publications (*n* = 20/44, 45.5%), as well as between publications and registrations (*n* = 16/21, 76.2%) or protocols (*n* = 6/6, 100%). Most inconsistencies within CT publications included a reduction from “parents or guardians” to “parental” consent, or from “legal” or “authorised” guardians to “guardians” only (*n* = 12/20, 60%). However, some CT publications (*n* = 8/20, 40%) also applied the terms interchangeably by, e.g. first using “caretaker” then “guardian”, or by switching between “parents” and “primary caregivers” or “mothers” [see Additional file 2, Table S2]. Inconsistencies between publications and registrations or protocols mostly included more or less specification of proxy decision-maker types and some included an interchangeable use of terms.

Table 4 shows the transparency levels of reported proxy decision-makers in publications and supplementary material. When considering only the CT publications, without supplementary material, and focusing on the most detailed description of proxy decision-makers within the main text of each publication, in half of all CT publications (50.0%), neither the type nor the number of proxy decision-makers was specified. In less than half CT publications (45.5%) transparency on the type of proxy decision-makers was extended, and only one publication provided a second level of extended transparency [31].

**Table 4:** Transparency levels of reported proxy decision-makers in main texts of CT publications and supplementary material

Transparency level	Proxy decision-maker description	All proxy decision-makers in CT publications and supplementary material		Most specific proxy decision-makers in main texts of CT publications	
		n	(%)	n	(%)
Basic	Informed consent from an undefined representative (e.g., parents, parents or guardians, caregivers, guardians, etc.)	31	(40.3)	22	(50.0)
Extended 1	Representative specified by number (e.g., at least one, each, one) or type (e.g., mothers, family, legal/authorised/identifiable guardian, etc.)	40	(51.9)	20	(45.5)
Extended 2	Representatives defined by age or competence (e.g., adult/of legal age, ability/capability to understand and give consent, a person with power of attorney)	6	(7.8)	1	(2.3)
**Total**		**77**	**(100.0)**	**43** ^a^	**(97.8)**

^a^ For one CT, the proxy decision-maker was only mentioned in the CT registration [62]

## Discussion

While some CT publications identified in our analysis indicated that children with minor parents might have been considered, none of the CT publications met a sufficient level of transparency to confirm whether such children were truly enrolled in the CTs and who consented on their behalf. Considering previously reported rates of children with minor parents recruited in individual CTs in LMICs, which ranged from 1.4–4.1% [6–8], 1051 to 3078 of the children in our review may have had minor parents unless they were excluded at screening based on parental age.

### Transparency on the recruitment of children with minor mothers

Publications of CTs recruiting children and their mothers as dyad may list maternal age among the eligibility criteria and the effective reasons for exclusion [31]. In other CT publications, however, maternal eligibility requirements were uncommon, perhaps because research focused on children independently from their mothers. Several CT publications included maternal data among the baseline data. These data contained maternal age (mean and standard deviation), years of education, or literacy levels [31, 40, 49, 60, 66–69]. The reporting of these aspects might aim at acknowledging some confounding factors pertinent for the study results. Two of the included CT publications also discussed possible relations between maternal data and study cooperation or the generalizability of study results [40, 68], which emphasizes the importance of including information on special population groups in CT publications. Hence, standard reporting of maternal age when including small children as CT participants and the additional provision of the maternal age thresholds (i.e. minimum and maximum age) is a straightforward way for researchers to increase transparency on the inclusion of children with minor mothers who are also the primary caregiver.

### Transparency on proxy decision-makers

In half of all CT publications, only a blanket statement was provided in the Methods section that IC was granted by four main proxy decision-maker types: “parents”, “parents or guardians”, “guardians”, or “caregivers”. The other half of the CT publications additionally specified subtypes of proxy decision-makers (e.g., mother, legal guardian, and primary caregiver) and their number (at least one, each, and one). In 11% of the CT publications (*n* = 5/44), CT registrations (*n* = 5/21, 23.8%) and corresponding protocols (*n* = 2/6, 33.3%) providing more transparency about the possible involvement of children with underage parents and the requirements for proxy decision-makers were referenced. Therein, consent was required from parents or guardians who were adults (or of legal age) or able (or capable) to understand study procedures and give consent [32, 33, 43, 63, 69]. However, since the corresponding CT publications contained only blanket statements that the consent was given by “parents or guardians”, the final procedure remained unclear.

Overall, the information on proxy decision-makers in CT publications was very brief and overlooked any difficulties or exceptions. This is surprising since previous literature shows that, for example, identifying suitable proxy decision-makers in paediatric CTs in LMICs can be challenging [73]. Moreover, depending on the policy, context or law applied, the meaning of the main proxy decision-maker types may vary and leave room for interpretation [see Additional file 2, Table S2]. Hence, there may be differences in whether falling into the category of “parent” or “caregiver” qualifies a person to be the proxy decision-maker, as they may still lack legal capacity. For example, in Malawi, biological fathers who are not married to a child’s mother do not automatically have direct “parental responsibility” rights in relation to the child [74]. These aspects require special attention in developing and reporting on the IC process. In the included CT publications, it remains uncertain if and how far such ethico-legal variation was considered. Confusion could also arise from applying the terms inconsistently throughout and between publications and their registrations or protocols, as found sometimes in this analysis. We, therefore, believe it is useful to establish standard operational definitions of possible proxy decision-maker types (e.g. guardians, legal guardians, legally acceptable representatives, caregivers) in international CT guidelines to facilitate a correct and coherent use of terminology.

Some of the publications referred to national CT guidelines they followed. However, again we could not find any particular statement on the case of children with minor parents. The general absence of information on children with minor parents in CT publications may suggest, first, that this case did not occur in these CTs, and, second, that researchers are not sensitised to the possibility of children having minor parents. In contrast, for example, several CT publications included additional details on other exceptional factors related to IC in LMICs, such as high illiteracy rates and providing oral consent with thumbprints, or the need for prior community consent. One publication also discussed the impact of the social and cultural background of the population on consent [57]. Third, authors of CT publications may focus more on reporting that IC was granted, regardless of how this was achieved.

Previous research showed that a detailed description of the consent process is uncommon in CT publications [75]. Consequently, the need to provide more details was debated [76]. Some critics question the practicality of adding succinct IC process descriptions, implying that it can be assumed that the adequacy of IC was evaluated and established by a competent ethics committee that has approved the CT [77]. While we agree that extensive IC descriptions in CT publications are not practical, given journals’ limited word count, it can also not be ignored that consent processes carry not only ethical but also scientific implications. IC procedures must be appropriate and tailored to the risks involved in each trial and may contribute to selection bias [78]. A description of how the IC procedure is handled for specific groups may, therefore, be useful for the overall picture of the CT.

For ethics committees to approve the appropriateness of IC procedures, these must be described in the CT protocol or reference must be made to applicable guidelines. Only publications can confirm what processes were actually applied. Publishing specific IC processes would not only help ensure the consent’s validity, but would also provide guidance for future researchers, prevent protocol deviations, and possibly highlight areas that need new guidance. Also, it can incentivise researchers to consider possible practical challenges early on, mitigating recruitment delays and consent withdrawals, and to strengthen reflections on risk-benefit analyses. We, therefore, recommend the general presentation of IC processes in CT publications and argue for the inclusion of evidence on exceptional IC cases. At a minimum, this should be described in an appendix to the publication in case of space limitations.

Ethical considerations, including consent, are currently not part of the CONSORT checklist [17]. Although the explanation and elaboration of CONSORT refers to IC and mentions that obtaining consent should be reported, it defers to journal instructions for specific ethical requirements [79]. The CONSORT adaptations for children, however, deem IC in paediatrics more complicated than for adults and propose consent related considerations to the checklist, such as reporting if assent was provided [19, 20]. This view reinforces our recommendations, as similar to paediatric CTs, consent procedures in CTs conducted in LMICs may require specific considerations meriting additional clarification in publications. In case a new CONSORT extension for paediatric CTs is developed, it should address ethical requirements and ask for an explicit description of exceptional IC situations for special population groups and of the solutions implemented.

The SPIRIT protocol development checklist already contains a requirement to specify how the IC should be obtained from participants [16]. Our review found that CT protocols that were likely developed after the implementation of SPIRIT provided increased transparency about the proxy decision-maker, emphasising the possible benefit of including consent requirements also in checklists for CT publications.

Furthermore, our study found that only a small number of publications provided information on the CT phase. However, a clear indication of the phase is useful to ascertain IC validity, as it is an indicator of research borne risks. It potentially has an impact on the operationalisation of the IC, including the selection and number of proxy decision-makers. The CONSORT explanation and elaboration paper also recognises that specifying the phase may be relevant in drug trials [79].

### Strengths and limitations

To our knowledge, this is the first systematic analysis of the recruitment and consent of children with minor parents in CT publications. Previous studies have shown that a small percentage of CT publications do not report the provision of consent [80, 81]. The search strategy was designed to identify articles that relate to consent and proxy decision-makers based on the information in titles and abstracts. If articles did not include such information in their titles and abstracts and were not otherwise linked to our topics of interest, they were probably omitted by the search.

In addition, CT publications that referred to other literature for more details on the research methodology, but did not specifically refer to “consent” or “permission” within their text, may have been missed. Our search strategy may have also overlooked some specific terms. For example, we searched for “adolescent pregnancy” but did not explicitly include the term “pregnant women”, which may have inadvertently excluded studies in which children of underage parents could be the focus of CTs with pregnant women.

Given these limitations, the extent of IC information provided in our sample of CT publications may not be generalizable. However, as young children in LMICs are particularly vulnerable research participants, this vulnerability may have led the respective researchers to report more cautiously than for typical research participants [80]. Therefore, it is also possible that our search strategy identified CT publications that provide above-average information on consent and the management of children with minor parents in research might be even less transparent than shown in our analysis, which underlines the importance of increasing standards in reporting.

Nevertheless, to follow-up on this research, we would recommend collecting and analysing a random sample of CT publications published from 2014 (after the CONSORT and SPIRIT guidelines were both introduced). This would also give the opportunity to extend the review to LMICs beyond SSA. Further strengths and limitations of the search strategy were previously published [9].

The authors acknowledge that directly involving collaborators based and working in research in the SSA region may have enriched the analysis and strengthen the conclusions of this work. The limitation of the study’s design is partially mitigated by the knowledge available from the researchers’ institution being involved in over 150 CTs executed in partnership and direct team discussions held with clinical research colleagues from the SSA region.

We recognize the general added value that engagement of stakeholders from the region can bring and recommend it to be included in future studies. Also, efforts to improve criteria for IC procedures, such as the CONSORT checklist, should include regional and cultural consultation, to avoid standardizing processes while overlooking their impracticality for specific situations.

## Conclusions

Despite the increased probability to encounter minor parents when recruiting children under 5 years of age in CTs in SSA countries, no CT publication in our analysis allowed us to ascertain whether such children were indeed included and who provided consent on their behalf. Transparency on the recruitment of children with minor parents could be increased when reporting additionally the minimum maternal age alongside maternal data provided in baseline data. Transparency on the person with the capacity to provide consent for children with minor parents could be increased by adding such information in the description of the IC process (in the publication or protocol) or by referring to relevant guidelines that address the issue in the specific country. In general, CT publications should include or reference exceptional IC procedures applied for special population groups and these ethical considerations should be required by the CONSORT checklist. A standardised terminology on proxy decision-maker types should be integrated in international CT guidelines and would facilitate correct and transparent consent processes for children in general and, more importantly, for children with minor parents. The process of developing international standards should include diverse experts from all geographical areas, in order to achieve comprehensive, sound and practical standards in complex contexts.

## Supplementary Information

**Additional file 1: Table S1.** Articles and conference abstracts not analysed due to missing access or not being found.

**Additional file 2: Table S2.** Examples of proxy decision-maker definitions and interpretations in various guidelines and contexts.

## Data Availability

The datasets used and/or analysed during the current study are available from the corresponding author on reasonable request.

## Abbreviations

SSA: Sub-Saharan Africa
CT: Clinical trial
CONSORT: Consolidated Standards of Reporting Trials
SPIRIT: Standard Protocol Items: Recommendations for Interventional Trials
EC: Ethics committee
IRB: Institutional review board
IC: Informed consent
EDCTP: European and Developing Countries Clinical Trial Partnership

## Footnotes

1 Proxy decision-maker is used throughout the manuscript in its colloquial form as employed by researchers and not in the legal sense. As such, it can refer to parents as (legal) surrogate decision-makers or formal and informal proxies, such as grandparents, other caregivers or court appointed guardians.
