# Supplementary material for: Transparent reporting of recruitment and informed consent approaches in clinical trials recruiting children with minor parents in sub-Saharan Africa: a secondary analysis based on a systematic review
Source: BMC Public Health. 2021 Jul 28;21:1473. doi: 10.1186/s12889-021-11079-y (PMC8318049; doi:10.1186/s12889-021-11079-y)
Supplement: Supplementary file 1 — Additional file 1: Table S1. Articles and conference abstracts not analysed due to missing access or not being found. [file 12889_2021_11079_MOESM1_ESM.docx]

### Additional file 1: Table S1 Articles and conference abstracts not analysed due to missing access or not being found

| **Author** | **Titel** | **Journal/Publisher** | **Type** |
| --- | --- | --- | --- |
| Baud et al. (2017) | Dosage regimen of biperiden to treat haloperidol-induced severe facio-troncular dystonic syndrome in children | Annals of Intensive Care | Conference Abstract |
| Bogie et al. (2015) | Non-invasive haemoglobin measurements for assessing anaemia in kenyan school children as part of an integrated school health and nutrition programme | Archives of Disease in | Conference Abstract |
| Bonsall et al. (2018) | HIV genotyping and phylogenetics in the HPTN 071 (PopART) study: Validation of a high-throughput sequencing assay for viral load quantification, genotyping, resistance testing and high-resolution transmission networking | Journal of the International AIDS Society | Conference Abstract |
| Clarke et al. (2012) | A new approach for malaria control in schools: Results of a randomized trial of intermittent parasite clearance | American Journal of Tropical Medicine and Hygiene | Conference Abstract |
| Close (2010) | The effect of probiotics in reducing the duration of acute infectious diarrhea in children: a literature review | International Journal of Probiotics & Prebiotics | Journal Article |
| Darabi et al. (2013) | The effect of vitamin D supplementation over asthma outcome | Iranian Journal of Allergy, Asthma and Immunology | Journal Article |
| Hamed (2007) | Early Delivery versus Expectant Management in Patients with Preterm Prelabour Rupture of Membranes at 34-37 weeks of Gestation. | The scientific J of El-minia Faculty of medicine | Journal Article |
| Kasule et al. (1995) | A clinical trial of Exluton, a progestogen only contraceptive pill containing 0.5mg lynestrenol amongst lactating Zimbabwean women | British Journal of Family Planning | Journal Article |
| Thornthwaite et al (2016) | Peroxybioflavonoids (MALSUP): A possible cure for severe cases of plasmodium falciparum malaria infection in Nigeria | European Journal of Immunology | Conference Abstract |
| Van Den Bent et al. (2017) | Evaluation of depatuxizumab mafodotin (ABT-414) in children with high grade glioma (HGG) and diffuse intrinsic pontine glioma (DIPG) | Neuro-Oncology | Conference Abstract |
| Watcha et al. (2010) | Validation of the baxter animated retching faces (BARF) scale for measuring nausea in children | Anesthesia and Analgesia | Journal Article |
| Weeks (2012) | Community-based use of misoprostol for pph prevention: Snapshot from a pilot study in Uganda documenting self-administration of misoprostol in a home delivery setting | International Journal of Gynecology and Obstetrics | Conference Abstract (Poster) |
