# Supplementary material for: Transparent reporting of recruitment and informed consent approaches in clinical trials recruiting children with minor parents in sub-Saharan Africa: a secondary analysis based on a systematic review
Source: BMC Public Health. 2021 Jul 28;21:1473. doi: 10.1186/s12889-021-11079-y (PMC8318049; doi:10.1186/s12889-021-11079-y)
Supplement: Supplementary file 2 — Additional file 2: Table S2. Examples of proxy decision-maker definitions and interpretations in various guidelines and contexts. [file 12889_2021_11079_MOESM2_ESM.docx]

### Additional file 2: Table S2 Examples of proxy decision-maker definitions and interpretations in various guidelines and contexts

| **Decision-maker** | **Legal interpretation** | **Examples of definitions** |
| --- | --- | --- |
| Parent | Decision-making authority (right to give permission for child participation) recognized by law | - Defined by national law: - "Parent" includes an adoptive parent (South African Children's Act 38 of 2005 [1]) - "Foster parent" (if there is no parent or guardian): per order of Children's Court (South African Children's Act 38 of 2005 [1]) - May be used in a broader context: "Throughout this report we use the term 'parents' to refer to one or more adults taking on this role of parental responsibility whether or not they have a biological connection with the child. In the UK context, for example, this will include legally appointed guardians and also many others, such as grandparents, who have acquired parental responsibility through a parental responsibility order or residence order." (Nuffield 2015 [2]) |
| Guardian | Decision-making authority exercised by proxy, typically with legal power when permission is delegated to a guardian who is legally appointed (recognized as legal representative, e.g. court-appointed). | - May be a short form for "legal guardian": - "Permission of a parent or legally authorized representative: The researcher must obtain the permission of at least one parent or guardian." (CIOMS 2016 [3]) - "[…] permission of a parent, legal guardian or other duly authorized representative." (CIOMS 2016 [3]) - "Parental (legal guardian) consent/permission: Expression of understanding and agreement by fully informed parent(s) or legal guardian to permit the investigator/sponsor of a clinical study to enrol a child in a clinical investigation." (ICH-E11 2017 [4]) - If "guardian" also equals "legal guardian" interpreted in the sense of "legally acceptable representative" as defined by the ICH-E6 2016 guideline, it relates to any individual authorized under applicable law to consent on behalf of the child, which also covers "parents". - May be defined by national law: "Guardian" (if there is no parent): either court-appointed OR as indicated by the parent in a will (South African Children's Act 38 of 2005 [1]). - May be used in a broader context: "In accordance with relevant national regulations, the permission of an immediate family member or other person with a close personal relationship with the individual must be sought […] in situations where a legally authorized representative is not available to allow for timely enrolment, researchers may obtain the permission of a representative who is socially accepted but not formally recognized before the law." (CIOMS 2016 [3]) |
| Caregiver | Informal or formal (when recognized as legal representative, e.g. court-appointed) decision-making authority exercised by proxy | - May be short for primary caregiver: "[…] a qualitative study was conducted to explore caregiver and community perceptions […] primary caregivers (i.e., mothers and fathers) of eligible infants […]" (Achieng et al. 2020 [5]). - May be defined by national law: "Caregiver" (if there is no parent, guardian, or foster parent) "[…] any person other than a parent or guardian, who factually cares for a child and includes - a) a foster parent; b) a person who cares for the child with the implied or express consent of a parent or guardian of the child; c) a person who cares for the child whilst the child is in temporary safe care; d) the person at the head of a child and youth care centre where a child has been placed; e) the person at the head of a shelter; f) a child and youth care worker who cares for a child who is without appropriate family care in the community; and g) the child at the head of a child headed household' (South African Children's Act 38 of 2005 [1]) |

**References**

1. Slack CM, Strode A. But is this really the ‘parent’or ‘guardian’? Practical strategies for consent to child research in South Africa. South African Journal of Bioethics and Law. 2016;9(1):35–8.

2. Nuffield Council on Bioethics. Children and clinical research: ethical issues. London; 2015.

3. CIOMS. International Ethical Guidelines for Health-related Research Involving Humans. Geneva; 2016.

4. ICH. ICH Harmonized Guideline. Addendum to ICH E11: Clinical Investigation of Medicinal Products in the Pediatric Population E11(R1). 2017.

5. Achieng F, Rosen JG, Cherop RY, Kariuki S, Hoffman SL, Seder R, et al. Caregiver and community perceptions and experiences participating in an infant malaria prevention trial of PfSPZ Vaccine administered by direct venous inoculation: a qualitative study in Siaya County, western Kenya. Malaria journal. 2020;19(1):226.
